# Evaluation of Aflatoxin M1 Effects on the Metabolomic and Cytokinomic Profiling of a Hepatoblastoma Cell Line

**DOI:** 10.3390/toxins10110436

**Published:** 2018-10-28

**Authors:** Silvia Marchese, Angela Sorice, Andrea Ariano, Salvatore Florio, Alfredo Budillon, Susan Costantini, Lorella Severino

**Affiliations:** 1 Unità di Farmacologia e Tossicologia—Dipartimento di Medicina Veterinaria e Produzioni Animali, Università degli Studi di Napoli “Federico II”, 80138 Napoli, Italy; silv.marchese@gmail.com (S.M.); andrea.ariano@unina.it (A.A.); salvatore.florio@unina.it (S.F.); 2 Unità di Farmacologia Sperimentale, Istituto Nazionale Tumori-IRCCS-Fondazione G. Pascale, 80131 Napoli, Italy; a.sorice@istitutotumori.na.it (A.S.); a.budillon@istitutotumori.na.it (A.B.)

**Keywords:** aflatoxin M1, metabolome, cytokinome, hepatoblastoma

## Abstract

Hepatoblastoma incidence has been associated with different environmental factors even if no data are reported about a correlation between aflatoxin exposure and hepatoblastoma initiation. Considering that hepatoblastoma develops in infants and children and aflatoxin M1 (AFM1), the aflatoxin B1 (AFB1) hydroxylated metabolite, can be present in mothers’ milk and in marketed milk products, in this study we decided to test the effects of AFM1 on a hepatoblastoma cell line (HepG2). Firstly, we evaluated the effects of AFM1 on the cell viability, apoptosis, cell cycle, and metabolomic and cytokinomic profile of HepG2 cells after treatment. AFM1 induced: (1) a decrease of HepG2 cell viability, reaching IC_50_ at 9 µM; (2) the blocking of the cell cycle in the G0/G1 phase; (3) the decrease of formiate levels and incremented level of some amino acids and metabolites in HepG2 cells after treatment; and (4) the increase of the concentration of three pro-inflammatory cytokines, IL-6, IL-8, and TNF-α, and the decrease of the anti-inflammatory interleukin, IL-4. Our results show that AFM1 inhibited the growth of HepG2 cells, inducing both a modulation of the lipidic, glycolytic, and amino acid metabolism and an increase of the inflammatory status of these cells.

## 1. Introduction

Hepatoblastoma is a common hepatic cancer of infancy and childhood, which occurs mainly in the first years of life [1]. Histologically, there are two sub-types of hepatoblastoma: (1) the epithelial type, including embryonal, fetal, combined embryonal and fetal, or small cell type and macrotrabecular; and (2) the epithelial/mesenchymal type with extra-mesenchymal elements [2]. Hepatoblastoma patients have elevated levels of α-fetoprotein (AFP), a useful marker of diagnosis and of treatment response [3]. Regarding hepatoblastoma biology, it is reported that CTNNB1 mutations and the Wnt pathway, which regulates this gene, are involved in the initiation of this cancer. Tan et al. (2005) knocked out CTNNB1 in a murine model, and demonstrated that partial hepatectomy induced hepatic cell proliferation and growth. This study evidenced that CTNNB1 abnormalities collectively account for most of the genetic defects in hepatoblastoma and its increased levels are present in almost all hepatoblastoma cases [4]. In recent years, the incidence of hepatoblastoma has been associated with genetic syndromes and parental environmental exposures, such as Beckwith–Wiedemann, very low birth weight, parental smoking, and parental exposure to metals used in soldering and welding, petroleum, or paints [5]. Hepatoblastoma development has also been correlated to fetal alcohol syndrome, oral contraceptive use during pregnancy, and maternal liver transplantation with immunosuppressive therapy [6]. 

In the literature, no data are reported about an association between aflatoxin B1 (AFB1) exposure and hepatoblastoma initiation, but only on liver cancer development after aflatoxins exposure through contaminated food [7]. The metabolism of AFB1 occurs in the liver in the presence of enzymes belonging to the cytochrome P450 (CYP450) superfamily [8]. Since it has been demonstrated that AFB1 exposure increased liver cancer risk, AFB1 was classified as a human carcinogen of Group 1 type; in fact, it favors the formation of DNA adducts that induce liver cancer development [9]. On the other hand, the hydroxylation of AFB1 produces aflatoxin M1 (AFM1), which is present in mammalian milk when the producing female is fed upon contaminated feedstuff. Since AFM1 was also found in the milk of lactating human mothers, it was indicated as a human carcinogen of Group 2B type. The maximum residue limits (MRLs) for AFM1 have been defined in milk [10,11,12].

In the case of AFB1, some data are known about its effects on a hepatoblastoma cell line (HepG2). Some authors recently demonstrated that AFB1 induced cytotoxicity and DNA damage in HepG2 cells [13]. Moreover, some authors evidenced that HepG2 cells treated with AFB1 presented high levels of miR-34a and a down-modulation of the Wnt/β-catenin signaling pathway [14]. Furthermore, another study demonstrated the formation of liver cancer stem cells (CSCs) after AFB1 treatment [15]. 

Regarding the effects of AFM1 on hepatoblastoma cells, there are only data related to its capacity to induce cytotoxicity and DNA damage in HepG2 cells [13]. Therefore, considering that hepatoblastoma develops in infants and children and AFM1 can be present in mother’s milk and in marketed milk products, in this study we decided to test the effects of AFM1 on a hepatoblastoma cell line (HepG2), not only in terms of cell proliferation, apoptosis, and cell cycle, but also through metabolomic and cytokinomic profiling to understand the effects of this aflatoxin on the metabolism and inflammatory status of these cells.

## 2. Results and Discussion

### 2.1. AFM1 Reduces Cell Proliferation and Induces a Block of the Cell Cycle in HepG2 Cells

**Figure 1:**
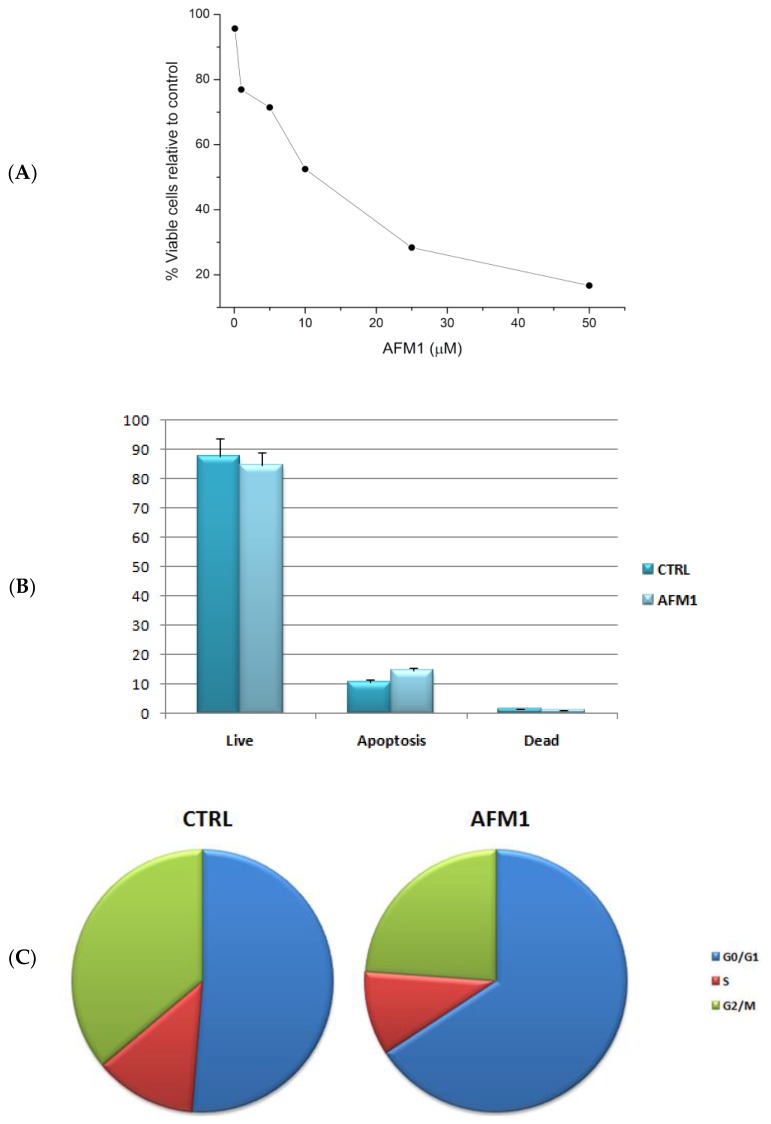
(**A**) Cell viability related to HepG2 cells after AFM1 treatment for 48 h. (**B**) Percentage of live, apoptotic, and dead cells (mean ± standard deviation) for HepG2 cells at IC_50_ concentration before (CTRL) and after (AFM1) 48 h of treatment. (**C**) Cell percentages in G0/G1, S, and G2/M phases (mean ± standard deviation) for HepG2 cells at IC_50_ concentration before (CTRL) and after (AFM1) 48 h of treatment.

To assess the concentration at which cell growth was inhibited by 50%, we performed sulforhodamine B (SRB) assay on a HepG2 cell line treated with different concentrations of AFM1. As shown in Figure 1A, incubation with AFM1 decreased the viability of HepG2 cells, and this reduction is significant relative to control cells (*p*-value <0.05). In particular, HepG2 cells reached IC_50_ at 9 µM after 48 h of treatment. Then, the capacity of AFM1 to induce apoptosis was evaluated at IC_50_ concentration after 48 h of treatment. No increase in the number of apoptotic cells was observed for HepG2 cells after AFM1 treatment (Figure 1B). Moreover, we evaluated whether there was a modulation of cell cycle in HepG2 cells after 48 h of incubation at IC_50_ concentration. Our results indicate that AFM1 is able to block the cell cycle in the G0/G1 phase (Figure 1C). 

### 2.2. Effects of AFM1 on the Metabolome of HepG2 Cells

**Figure 2:**
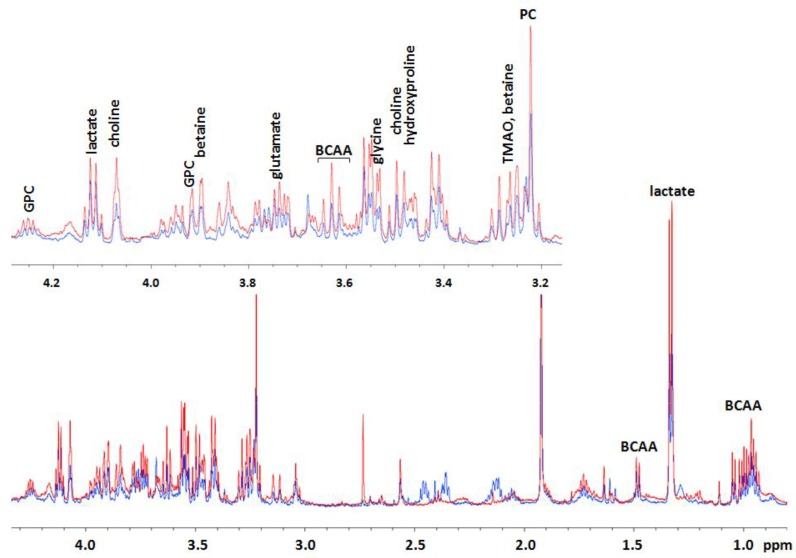
Superposition of mono-dimensional Nuclear Magnetic Resonance (^1^H-NMR) spectra of the polar fractions obtained from treated (red spectrum) and untreated (blue spectrum) HepG2 cells in the spectral region from 0.8 to 4.3 ppm, with a magnification of the spectral region between 3.15 to 4.3 ppm. We report the spectral assignments of the metabolites that significantly increased after treatment. In detail, GPC: Glycerophosphocholine and BCAA: branched-chain amino acids.

**Figure 3:**
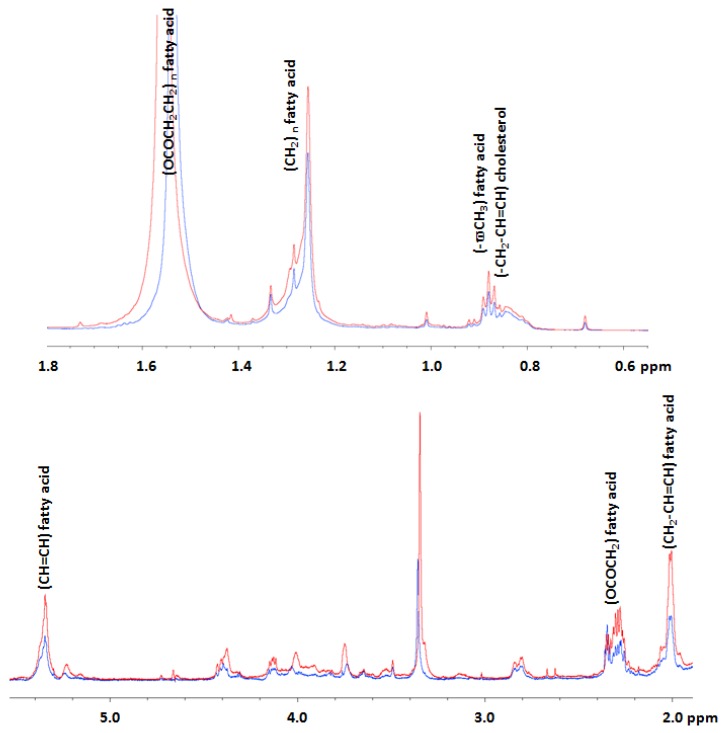
Superposition of mono-dimensional Nuclear Magnetic Resonance (^1^H-NMR) spectra of the lipidic fractions obtained for treated (red spectrum) and untreated (blue spectrum) HepG2 cells in the spectral regions from 0.55 to 1.8 and from 2 to 5.5 ppm. We report the spectral assignments of the lipids that significantly increased after treatment.

In order to better understand the effects of AFM1, a metabolomic analysis was conducted on HepG2 cells treated with AFM1, and compared to untreated cells. The analysis of the obtained spectra for HepG2 cells evidenced: (1) for the polar fractions, the presence of spectral signals of amino acids, lactate, glucose, formiate, choline, phosphocholine, and glycerophosphocoline (Table 1 and Figure 2); and (2) for the lipidic fractions, the presence of spectral signals related to cholesterol, fatty acids, phospholipids, triglycerides, choline, and phosphatidylcholine (Table 1 and Figure 3).

**Figure 4:**
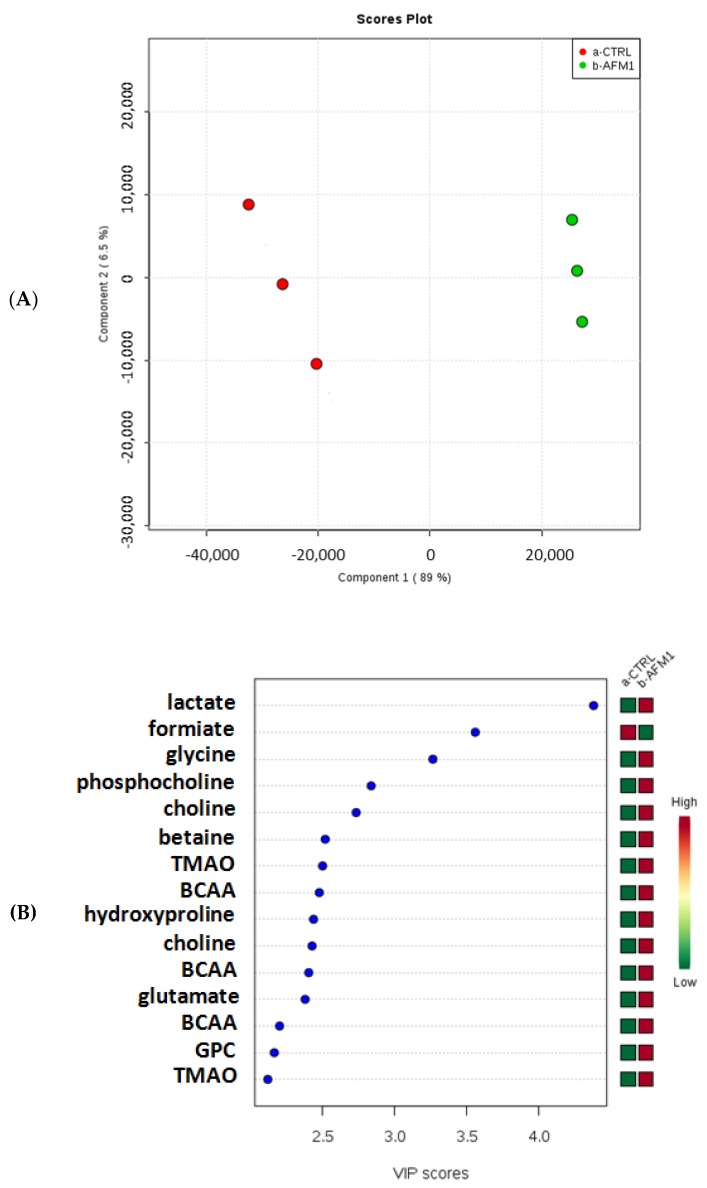
Partial least squares-discriminant analysis (PLS-DA) (**A**) and variable importance in projection (VIP) (**B**) plots related to the polar fraction of the HepG2 cell line treated with AFM1 compared to untreated cells.

**Figure 5:**
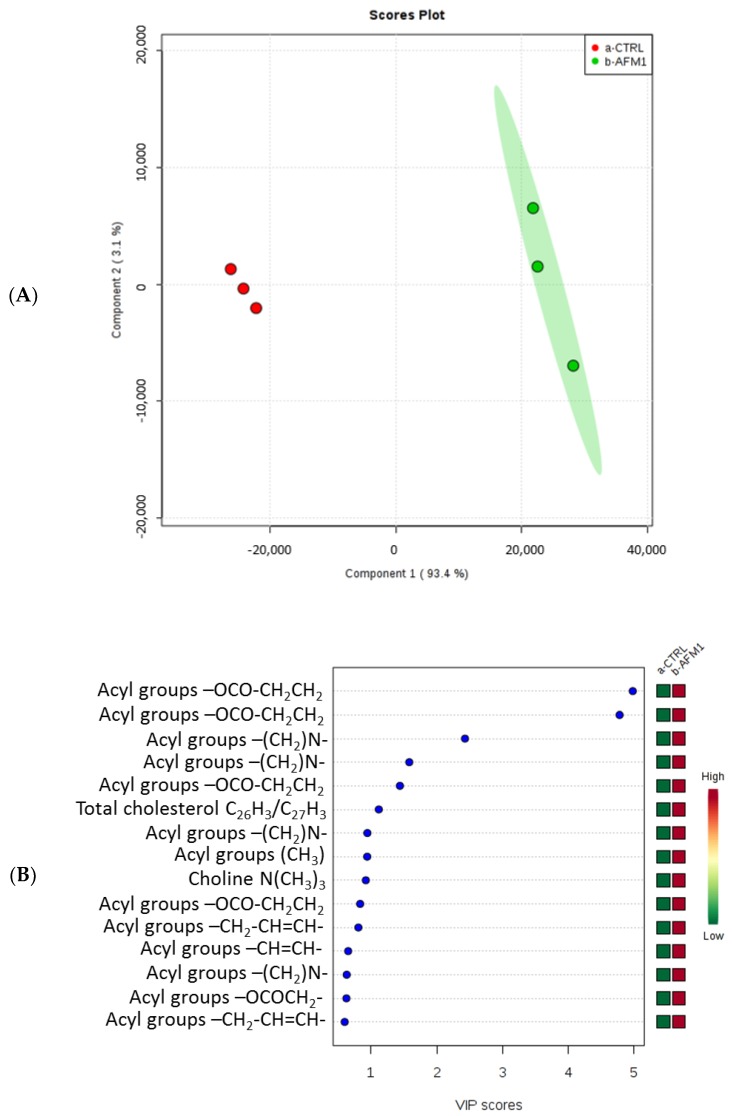
Partial least squares-discriminant analysis (PLS-DA) (**A**) and variable importance in projection (VIP) (**B**) plots related to the lipidic fraction of the HepG2 cell line treated with AFM1 compared to untreated cells.

Multivariate statistical analysis evidenced a marked difference between treated samples and control samples (Figure 4A and Figure 5A). Moreover, the variable importance in projection (VIP) scores plot showed a modulation of metabolites involved in different pathways and known to be important in cancer development and growth (Figure 4B and Figure 5B). Specifically, the levels of acyl groups of fatty acids and cholesterol, as well as those of lactate, glycine, choline, phosphocholine (PC), glycerophosphocholine (GPC), betaine, trimethylamine N-oxide (TMAO), hydroxyproline, branched-chain amino acids (BCAA), and glutamate, were increased in treated HepG2 cells, whereas the levels of formiate were decreased after AFM1 treatment. 

Therefore, the metabolomic analysis evidenced that AFM1 induces a modulation of the lipidic, glycolytic and amino acid metabolism. Specifically, the functional enriched analysis evidenced that the significant metabolites are involved in the following pathways: glycine, BCAAs, and glutamate in aminoacyl-tRNA biosynthesis; TMAO, glycine, and formiate in methane metabolism; glutamate, glycine, and formiate in nitrogen metabolism; choline, PC, and GPC in glycerophospholipid metabolism; choline, betaine, and glycine in glycine, serine, and threonine metabolism; lactate and formiate in pyruvate metabolism; lactate and BCAAs in propanoate metabolism; glutamate and glycine in glutathione metabolism; glycine and cholesterol in primary bile acid biosynthesis.

However, as is evident in Figure 2, in the shift region between 2.0 and 2.5 ppm there are some proton signals that are higher in untreated cells. We tried to perform J-resolved (JRES) NMR experiments to assign these signals but we could not distinguish these signals; therefore, a precise assignment was not possible. However, we suppose that these signals can refer to glutathione, as in the literature some authors evidenced that mice injected with *Toxoplasma gondii* tissue and fed aflatoxin exhibited a significant reduction in glutathione levels [16].

### 2.3. Evaluation of the Cytokine Levels of HepG2 Cells after AFM1 Treatment

Cytokines are involved in all inflammatory processes, and in cancer initiation and progression. In 2010, our group defined the term “cytokinome” to consider all the cytokines present in a given biological system. At present, it is possible to measure contemporaneously the concentrations (expressed in pg/mL) of many cytokines using multiplex ELISA-based immunoassay [17]. Hence, we decided to evaluate the levels of a panel of 27 cytokines in HepG2 cellular supernatants after treatment with IC_50_ of AFM1 for 48 h. Untreated cells were used as control. As shown in Table 2, the levels of IL-6, IL-8, and TNF-α increased after treatment, whereas those of IL-4 decreased. 

## 3. Discussion

Given that AFM1 is considered a hazard for human health—and in particular for children, due to its presence in milk and milk-derived products—and taking into account that liver is the main target organ of aflatoxins, we decided to focus our experiments on the HepG2 cell line, as it is obtained from the epithelial hepatoblastoma tissue of a 15 year old white male. These cells are negative for the Hepatitis B virus (HBV) and present wild-type p53 status [18], a loss of the chromosome 4q3 region, and trisomies 2 and 20 [19]. Moreover, HepG2 cells also have an exon 3 deletion of CTNNB1 [19], which is mutated in more than 85% of hepatoblastomas [20]. Furthermore, the cells also show also a low expression of cytochrome P450 (CYP)-metabolizing enzymes; remembering that AFM1 cytotoxicity might be exerted even without CYP activation [8], these cells therefore represent a good cellular model to study in vitro the effects of AFM1. 

Hence, in this work we tested the effects of AFM1 on cell proliferation, apoptosis induction, and cell cycle modulation of HepG2 cells. These studies evidenced that AFM1 was able to reduce cell proliferation cells reaching IC_50_ at 9 µM after 48 h of treatment (Figure 1). This concentration is certainly high enough if we consider what should be the mother intake to excrete this amount. In fact, some authors evaluated AFM1 levels in the breast milk of Egyptian mothers, and reported that: (1) AFM1 levels in the milk samples ranged from 8 pg/mL to 64 pg/mL; (2) in milk daily assumption considering 500 mL/day AFM1 represents between 0.09 and 0.43% of dietary intake; and (3) the daily AFB1 intake of the mothers should be a maximum of 6.7 ng/kg bw/day [21]. In a previous study conducted in Tanzania, it was shown that AFM1 in breast milk samples ranged from 0.01 to 0.55 ng/mL and the related exposure of the mothers was a maximum of 66.79 ng/kg bw/day [22]. Hence, if we consider these data, our IC_50_ obtained for HepG2 cells should correspond to high concentration, however it is necessary to underline that it is not often suitable to compare data obtained in vitro on cell lines and those obtained by in vivo studies. For in vitro studies, we must understand in a short time—in our case after only 48 h—what can be the effect of a mycotoxin on cell proliferation and metabolism, whereas for in vivo studies the exposure is daily. Moreover, in the case of cancer cells that have an accelerated growth and metabolism, in order to verify in a short time the effects of a molecule it is often necessary to increase the dose or repeat the treatment with a lower dose for further days or weeks in order to simulate the daily exposure. Another point to consider is that AFM1 could also be derived from mammalian milk fed upon contaminated feedstuff, and hence the amount of AFM1 arriving to infants/children can increase and AFM1 effects can derive from the sum of different exposure sources by a synergic and additive process. Moreover, in humans and mammals, the mycotoxin exposure risk derives from small doses repeated over time, which is certainly not reproducible by in vitro models. The cell lines represent “studying models” to investigate the molecular mechanisms that are modulated, activated, or inhibited by treatments, and are not able to define in more detail the kinetics.

Additionally, AFM1 was able to induce a block of the cell cycle in the G0/G1 phase, but not to induce apoptosis (Figure 1). These findings are in accordance with data recently published by Zheng et al. (2018), who reported how AFM1 was able to reduce cell proliferation and promote DNA damage in HepG2 cells [13]. The mycotoxins AFB1 and AFM1 have also been previously tested on human intestinal Caco-2 cells. The obtained data showed that AFB1 and AFM1 blocked the growth of both differentiated and undifferentiated cells, increased the concentration of lactate dehydrogenase (LDH), and induced a genetic damage [23,24]. Our data are also in agreement with another paper, in which the effects of AFM1 and AFB1 were evaluated on lymphoblastoid Jurkat T-cell line, demonstrating that these mycotoxins were able to significantly inhibit cell proliferation but not to activate apoptosis [25].

Subsequently, we additionally conducted a metabolomic analysis on HepG2 cells treated with AFM1, compared to untreated cells, by ^1^H-NMR, in order to understand the effects of AFM1 on cell metabolism. Our studies evidenced that in treated HepG2 cells there was a decrease in the levels of formiate and an increase in the levels of acyl groups of fatty acids and cholesterol, as well as those of lactate, glycine, choline, phosphocholine (PC), glycerophosphocholine (GPC), betaine, trimethylamine N-oxide (TMAO), hydroxyproline, branched-chain amino acids (BCAA), and glutamate (Figure 4 and Figure 5), suggesting a modulation of the lipidic, glycolytic, and amino acid metabolism.

It is well known that cancerous cells show an elevated glucose uptake and that, according to what is called the Warburg effect, such cells reprogram their metabolism, diverting the conversion of glycolysis-derived pyruvate from acetyl-CoA to lactate synthesis either in normoxic or hypoxic conditions [26]. This observation is concordant with the data published in 2011 which showed that lactate is able to stimulate angiogenesis. Some authors reported that lactate can induce an increase of autocrine NF-κB/IL-8 pathway and stimulate both cell migration and tube formation in both in vivo and in vitro studies [27]. Hence, the higher levels of lactate observed after AFM1 treatment suggests the enhancement of glycolytic pathway and disruption of TCA cycle.

Considering that formiate is produced by pyruvate and coenzyme A in the presence of pyruvate formiate lyase enzyme, we can suggest that after AFM1 treatment the pyruvate is consumed to form lactate and, hence, the conversion from pyruvate to formiate is slowed down. This explains why we observed lower levels of formiate after AFM1 treatment.

In accordance with our data, choline, PC, and GPC are widely reported to be increased in tumorous tissues [28,29,30]. Since choline is a component of PC and GPC, which are fundamental elements of phospholipids for cell membrane synthesis, increased levels of choline and its metabolites have been linked to membrane disruption, liver damage and, hence, to cancer development. In fact, some authors found higher levels of choline, PC, and GPC in rat liver exposed to AFB1, and demonstrated the loss of structural cell membrane integrity [31].

However, we must remember that: (1) betaine is a compound with one positively charged and one negatively charged functional group that serves as organic osmolyte and is used by cells for protection against osmotic stress; and (2) TMAO is another osmolyte obtained as a product of the oxidation of trimethylamine which is derived from choline. Hence, the high levels of these two osmolites are strongly correlated between them, and with high levels of choline, PC, and GPC suggesting that the increase of their levels represents a cellular reply aimed to protect osmolarity and block cell damage [30].

Proline metabolism in hepatocellular carcinoma (HCC) tissues is characterized by accelerated consumption of proline and accumulation of hydroxyproline. Intracellular hydroxyproline can also originate from procollagen digested by prolidase, the only known cytosolic dipeptidase, which breaks down dipeptides to yield free proline and hydroxyproline. Furthermore, hydroxyproline may function as a regulatory hub in low-oxygen conditions and stabilize hypoxia-induced factor-1α (HIF1α) in response to hypoxia. Recently some authors evidenced an accumulation of hydroxyproline in two HCC cohorts that positively correlated with AFP levels and poor prognosis. Importantly, they showed that hydroxyproline promoted angiogenesis supporting a HIF1α dependent mechanism [32]. Hence, our results are in agreement with these data.

The serine/glycine pathway represents an important point for cell survival, providing building blocks for proteins, nucleic acids, and lipid synthesis and regulating cellular anti-oxidative capacity [33]. Specifically, serine is a precursor of the nonessential amino acids glycine and cysteine, and glycine is in turn a precursor of porphyrins and is also incorporated directly into purine nucleotide bases and into glutathione (GSH). The conversion of serine to glycine, catalyzed by serine hydroxymethyltransferase (SHMT), donates a one-carbon unit to tetrahydrofolate to produce 5,10-methylenetetrahydrofolate (CH2-THF). Higher levels of glycine, as well as of glutamate, lactate, and choline were found in tissues of HCC patients by high resolution magic angle spinning (HR-MAS) NMR spectroscopy [28]. Thus, concerning the higher levels of glutamate found in HepG2 cells after AFM1 treatment, it is necessary to remember that many cancer cells have a high dependency on glutamine for their growth and survival. In fact, when glutamine enters into the cell via its transporter, it is converted to glutamate in the presence of glutaminase enzyme, and the glutamate is then converted to α-ketoglutarate and channeled into the TCA cycle [34].

On the other hand, the branched-chain amino acids (BCAAs) comprise valine, leucine, and isoleucine, being three essential amino acids. The BCAA metabolism in cancer has been recently reviewed, highlighting the involvement of these amino acids in the growth of different tumor cell types [35]. In fact, an upregulation of BCAA-associated metabolic enzymes has been reported in different tumor types, including HCC [36]. Moreover, leucine resulted in upregulation also in HCC samples as determined by HR-MAS NMR spectroscopy [28]. Overall, the high levels of BCAAs and glycine found in treated HepG2 cells compared to untreated cells are in good agreement with the results obtained in AFB1-exposed mice [31]. Hence, we can suggest that AFM1 can cause the disruption of hepatic amino acid metabolism by increasing the levels of these amino acids in male chicks [37] and rats [38].

Concerning the high levels of cholesterol and fatty acids in HepG2 cells after AFM1 treatment, we underline that our results agree with other published studies in which acute exposure to AFB1 increased the levels of liver cholesterol [39] and other hepatic lipids [31]. Moreover, it has also been demonstrated that fatty acid synthase is up-expressed in liver cancer and promotes the synthesis of endogenous fatty acids that supply energy used for the cancer progression [40]. 

Finally, to understand the effects of AFM1 on the inflammatory status of HepG2 cells, we decided to evaluate a large panel of cytokines in HepG2 cellular supernatants after IC_50_ treatment at 48 h. Our results evidenced that the levels of IL-6, IL-8, and TNF-α increased after treatment, whereas those of IL-4 decreased (Table 2). Considering that IL-4 is an anti-inflammatory interleukin, and IL-6, IL-8, and TNF-α are pro-inflammatory cytokines, our results evidenced that AFM1 induces an increase of the inflammatory status of HepG2 cells. These findings agree with previous studies. For instance, in Jurkat T-cells, AFB1 and AFM1 were able to block cell proliferation and increase IL-8 levels [25], whereas in vivo studies of pigs exposed to AFB1 showed increased levels of IL-6 [41]. Additionally, AFB1 treatment induced the decrease of IL-4 levels and the increase of TNF-α levels on splenic lymphocytes [42]. Furthermore, in humans exposed to AFB1 the levels of some pro-inflammatory cytokines increased [43].

## 4. Conclusions

Considering that hepatoblastoma develops in infants and children, and AFM1 can be present in mothers milk and in marketed milk products, our aim was to evaluate if AFM1 affects cell growth, metabolism, and inflammatory status of HepG2, a hepatoblastoma cell line. Our results demonstrated that AFM1 induced both the inhibition of cell growth and a block of cell cycle in the G0/G1 phase, but no increase of the number of apoptotic cells. Moreover, the analysis of the metabolomic profiling on HepG2 cells by 1H-NMR approach evidenced an increase in the levels of acyl groups of fatty acids and cholesterol, as well as those of lactate, glycine, choline, PC, GPC, betaine, TMAO, hydroxyproline, BCAAs, and glutamate, and a decrease of formiate levels in treated HepG2 cells. Moreover, after AFM1 treatment, the concentrations of IL-6, IL-8, and TNF-α were increased, and the concentration of IL-4 was decreased, in HepG2 cells. 

Therefore, overall data evidenced that AFM1 was able to induce in HepG2 cells an increased synthesis of lipids and amino acids, membrane damage, and the enhancement of the glycolytic pathway and inflammatory status. Hence, we can conclude that the possible presence of AFM1 in mother’s milk or in marketed milk products represents a topic to consider during hepatoblastoma progression and treatment.

## 5. Materials and Methods 

### 5.1. Cell Culture

Human hepatoblastoma (HepG2) cells were cultured and expanded in culture medium DMEM (Dulbecco’s Modified Eagle’s Medium, Lonza, Verviers, Belgium) supplemented with Penicillin/Streptomycin 100x (Euroclone, Devon, UK), 10% fetal bovine serum (FBS, Invitrogen, Camarillo, CA, USA), and non-essential amino acids 100x (Invitrogen, Camarillo, CA, USA) and Glutamax 100x (Invitrogen, Camarillo, CA, USA). The cells were kept at 37 °C in an incubator (5% CO_2_ and 95% air).

### 5.2. Cell Treatment and Cell Proliferation Assay

Colorimetric assay with sulforhodamine B (SRB, Sigma-Aldrich, St. Louis, MO, USA) was used to assess HepG2 proliferation before and after AFM1 treatment. A stock solution of AFM1 at a concentration of 1 mM was prepared in dimethyl sulfoxide (DMSO Sigma-Aldrich, St. Louis, MO, USA) arriving to a final DMSO concentration of lower than 0.1% by serial dilutions. A total of 2 × 10^3^ cells per well were plated and allowed to attach for 24 h. Cells were then stimulated with 0.1, 1, 5, 10, 25, and 50 µM concentrations of AFM1 (this concentration range was selected on the basis of the literature [21,22]). After 48 h of treatment, cells were fixed with trichloroacetic acid (Sigma-Aldrich, St. Louis, MO, USA) at 4°C for 1 h and then stained for 30 min with 0.4% (wt/vol) SRB dissolved in 1% acetic acid. Then, the cells were solubilized with 10 mM Tris base solution (pH=10.5). We measured the absorbance at 540 nm by a fluorometric assay (Bio-Rad, Hercules, CA, USA; Microplate Reader). Dose–response curves were performed to assess IC_50_.

### 5.3. Apoptosis Evaluation at IC_50_ Concentration after 48 h of Treatment 

The number of live, apoptotic, and dead cells was evaluated by Annexin V and Dead Cell Assay kit (Merck Millipore, Darmstadt, Germany) and Muse™ Cell Analyzer (Merck Millipore) using 3 × 10^5^ untreated and treated HepG2 cells and a protocol already reported in our recent paper [44].

### 5.4. Cell Cycle Assay at IC_50_ Concentration after 48 h of Treatment

To evaluate the number of cells in the different phases of the cell cycle, 1 × 10^6^ untreated and treated HepG2 cells were counted by Muse™ Cell Cycle Assay containing propidium iodide and nuclear DNA intercalating stain RNAse A. Cells were washed with phosphate buffered saline (PBS) and centrifuged. The obtained cell pellet was re-suspended in 1 mL of ice-cold 70% ethanol and then frozen at −20° C. After 24 h, the cells were washed with PBS and suspended at room temperature and in the dark for 30 min with 200 μL of Muse™ Cell Cycle Reagent. Then, the cells were counted.

### 5.5. Extraction of the Polar and Lipidic Fractions in Untreated and Treated HepG2 Cells

HepG2 cells were plated and treated with AFM1 at a concentration before IC_50_ (5 μM). Cell pellets (2 × 10^6^) related to untreated and treated HepG2 cells were washed twice in PBS and deuterated water (PBS-D_2_O) and frozen at −80 °C. The cells were subsequently suspended in 520 µL of H_2_O, 700 µL of methanol, and 700 µL of chloroform, sonicated for 30 s, shaken in ice on an orbital shaker for 10 min, and then centrifuged at 10,000 rpm at 4°C for 10 min, as reported in our recent paper [44]. 

Finally, the polar and lipidic phases were collected separately and evaporated. 

### 5.6. 1H-NMR Metabolomic Analysis of the Cellular Fractions

^1^H spectra of the cellular polar and lipidic fractions were acquired at 300 K by a 600 MHz Bruker spectrometer equipped with a TCI cryoprobe. The lipidic fractions were dissolved in 700 µL of CDCl_3_, and the polar fractions were dissolved in 630 µL of PBS-D_2_O and 70 µL of sodium salt of 3-(trimethylsilyl)-1-propanesulfonic acid (1% in D_2_O) used as the internal standard. 

The protocols used for the spectra acquisition and the proton signals assignments have already been reported in Nittoli et al. (2018) [44] and Ruocco et al. (2018) [45].

### 5.7. Statistical and Pathway Analysis

AMIX package (Bruker, Biospin GmbH, Rheinstetten, Germany) was used to integrate the 0.50–8.60 ppm spectral regions of the ^1^H-NMR spectra by buckets of 0.02 ppm and to exclude the water resonance regions (4.5–5.2 ppm) in the case of polar fractions during the following analysis. All the bucketed regions were normalized to the total spectrum area using Pareto scaling. To compare the spectra obtained from the polar and lipidic fractions obtained for HepG2 cells after AFM1 treatment compared to untreated cells, we performed partial least squares-discriminant analysis (PLS-DA) and VIP plot by Metabo Analyst tool that was used also for pathway analysis [46]. 

### 5.8. Bio-Plex Assay

To evaluate the cytokine levels on supernatants of HepG2 cells, untreated and treated at IC_50_ for 48 h, we used Bio-Plex Pro Human Cytokine 27-Plex Immunoassay and a Bio-Plex array reader (Luminex, Austin, TX, USA). The levels of the following cytokines were measured using a standard curve, generated by the software provided by the manufacturer (Bio-Plex Manager Software, version 5.0, Luminex, Austin, TX, USA): IL-1β, IL-1ra, IL-2, IL-4, IL-5, IL-6, IL-7, CCL2, CCL11, CXCL10, CXCL8, IFN-γ, IL-9, IL-10, IL-12 (p70), IL-13, IL-15, IL-17, basic FGF, G-CSF, GM-CSF, MIP-1α, MIP-1β, PDGF-ββ, RANTES, TNF-α, and VEGF. 

## Figures and Tables

**Table 1:** List of metabolites and ^1^H chemical shift (ppm) assigned on HepG2 cells.

Metabolites	Chemical shift (ppm)
Acetate	1.91
Alanine	1.48; 3.75
Arginine	1.68; 1.9; 3.26; 3.76
Betaine	3.25; 3.89
Choline	3.19; 3.51; 4.06
Formiate	8.44
Glucose	3.23; 3.39; 3.46; 3.52; 3.73; 3.82; 3.88; 4.63; 5.22
Glutamate	2.04; 2.12; 2.34; 3.75
Glutamine	2.13; 2.45; 3.77
Glycerophosphocholine (GPC)	3.20; 3.36;3.90; 4.28
Glycine	3.54
Histidine	3.16; 3.23; 7.09; 7.9
Hydroxyproline	2.14; 2.42; 3.36; 3.46; 4.33
Isoleucine	0.93; 0.99; 1.24; 1.46; 1.97; 3.66
Lactate	1.33; 4.11
Leucine	0.96; 1.72; 3.72
Lysine	1.46; 1.71; 1.89; 3.02; 3.74
Malate	2.36; 2.66; 4.29
Phenylalanine	3.19; 7.32–7.42
Phosphocholine (PC)	3.21; 3.58; 4.17
Proline	1.99; 2.06; 2.34; 3.33; 3.41; 4.12
Pyruvate	2.46
Threonine	1.32; 3.58; 4.24
Trimethylamine N-oxide (TMAO)	3.25
Tyrosine	3.02; 3.17; 3.92; 6.9; 7.2
Valine	0.97; 1.04; 2.28; 3.60
Cholesterol C_18_H_3_	0.67
C_26_H_3_,C_27_H_3_	0.88
C_19_H3	1.01–1.03
C_3_H	3.5
C_6_H	5.37
Fatty acid residues ω-CH_3_	0.89
(CH_2_)_n_	1.3
-COCH_2_-CH_2_	1.6
-CH_2_-CH=	2.04
-CO-CH_2_	2.3
-CH=CH-CH2-CH=CH	2.76
-CH=CH	5.36
Phosphatidylcholine (POCH2)	4.33–4.43
Phospholipids (-CH_2_-NH_2_)	3.11–3.14
(-CH_2_-N-(CH_3_)_3_)	3.33
Triglycerides C_1_H	4.15
C_3_H	4.29
C_2_H	5.25

**Table 2:** Fold change evaluated considering the concentrations of each cytokine in HepG2 after AFM1 treatment, compared to untreated cells. In particular, anti-inflammatory cytokines whose concentrations decreased after treatment are listed in italic and underlined and the pro-inflammatory cytokines whose concentrations increased after treatment are listed in bold.

Cytokines	Ratio (HepG2-AFM1 vs CTRL)
**PDGF-ββ**	0.94
**IL-1β**	1.04
**IL-1ra**	1.24
**IL-2**	0.92
**IL-4**	*0.68*
**IL-5**	0.98
**IL-6**	**1.30**
**IL-7**	0.92
**IL-8**	**1.73**
**IL-9**	0.96
**IL-10**	0.95
**IL-12**	0.90
**IL-13**	1.00
**IL-15**	0.90
**IL-17**	0.88
**Eotaxin**	0.96
**FGF basic**	0.96
**G-CSF**	1.00
**GM-CSF**	0.86
**IFN-γ**	0.89
**IP-10**	0.95
**MCP-1**	0.92
**MIP-1α**	0.96
**MIP-1β**	0.86
**RANTES**	0.90
**TNF-a**	**1.32**
**VEGF**	1.00
